# The Gene Encoding Dihydroflavonol 4-Reductase Is a Candidate for the *anthocyaninless* Locus of Rapid Cycling *Brassica rapa* (Fast Plants Type)

**DOI:** 10.1371/journal.pone.0161394

**Published:** 2016-08-22

**Authors:** Douglas L. Wendell, Anoumid Vaziri, Gurbaksh Shergill

**Affiliations:** Department of Biological Sciences, Oakland University, Rochester, Michigan, United States of America; New Mexico State University, UNITED STATES

## Abstract

Rapid cycling *Brassica rapa*, also known as Wisconsin Fast Plants, are a widely used organism in both K-12 and college science education. They are an excellent system for genetics laboratory instruction because it is very easy to conduct genetic crosses with this organism, there are numerous seed stocks with variation in both Mendelian and quantitative traits, they have a short generation time, and there is a wealth of educational materials for instructors using them. Their main deficiency for genetics education is that none of the genetic variation in RCBr has yet been characterized at the molecular level. Here we present the first molecular characterization of a gene responsible for a trait in Fast Plants. The trait under study is purple/nonpurple variation due to the *anthocyaninless* locus, which is one of the Mendelian traits most frequently used for genetics education with this organism. We present evidence that the *DFR* gene, which encodes dihyroflavonol 4-reductase, is the candidate gene for the *anthocyaninless* (*ANL*) locus in RCBr. *DFR* shows complete linkage with *ANL* in genetic crosses with a total of 948 informative chromosomes, and strains with the recessive nonpurple phenotype have a transposon-related insertion in the *DFR* which is predicted to disrupt gene function.

## Introduction

Rapid cycling *Brassica rapa* (RCBr), more commonly known as Wisconsin Fast Plants, is a widely used organism in science education. They are used at both K-12 and college levels as a model system for topics including growth and development, ecology, physiology, reproduction, and genetics. They have a six week generation time, a petite growth habit, easily grown in conventional potting soil, and do not require greenhouses or growth chambers. They thrive at room temperature under ordinary fluorescent lights, although the light must be constant and intense [1]. Their other major strength is that a huge volume of educational modules and support material for teachers has been developed by the Wisconsin Fast Plants Program [2].

Fast Plants are an appealing system for teaching genetics. Because *Brassica rapa* are self-incompatible for pollination, a student at any level can conduct a genetic cross by simply using a cotton swab to transfer pollen between plants [1]. A wide collection of seed stocks is available for experimentation in both Mendelian and quantitative traits, and these are readily available to educators from Carolina Biological Supply (Burlington, NC, USA) or from the Rapid Cycling Brassica Collection [2]. Dozens of Mendelian traits have been identified but the most frequently used are anthocyanin expression (purple *versus* non-purple stem), leaf color (green *versus* yellow-green), and trichome (hairless *versus* presence of hairs). Pigmentation and trichome density also exhibit quantitative variation [3]. Among plants that express anthocyanin pigments there is great variation in the intensity of the pigmentation and this variation is controlled by both polygenic inheritance and environment [4,5]. However, despite their widespread use in genetics education, none of the heritable traits in Fast Plants strains have been characterized at the molecular level.

The molecular genetics of anthocyanin expression has been studied in many plant systems. Anthocyanins are a group of flavonoid pigments that give color to flowers, fruit, stems, and leaves to varying degrees in many plant species. They play diverse roles including, but not limited to, attracting pollinators and frugivores, protection from herbivores, protection against ultraviolet light, and scavenging free radicals [6,7]. Numerous “anthocyanin genes” have been identified in plants, including the Brassicaceae family. Anthocyanin genes fall in to two broad categories: “structural genes” that encode enzymes in the biosynthetic pathways leading to formation of anthocyanins and “regulatory genes” that encode transcription factors needed for activation of the structural genes [8,9].

Here we present the first molecular characterization of a gene responsible for a trait in Fast Plants. We report here that non-purple strains of RCBr have a transposon-related insertion in the fourth exon of the gene encoding dihydroflavonol-4-reductase (DFR). This mutation shows complete linkage with the *anthocyaninless* (*anl*) locus indicating that *anl* and *DFR* are most likely the same gene.

## Materials and Methods

### Plant Strains

The rapid cycling *Brassica rapa* (Fast Plants type) strains Purple Stem, Hairy (*ANL*/*ANL*) and Non-Purple Stem, Yellow-Green Leaf (*anl*/*anl*) were obtained from Carolina Biological Supply (Burlington, NC). Purple (*ANL*/*ANL*) strain DWRCBr76 was reported previously [10]. Non-purple (*anl*/*anl*) strain DWRCBr57 was derived from DWRCBr53 [10] with selection for homozygosity of DNA markers on chromosome A09.

### Genetic Marker Developments

*Brassica rapa* genome sequence was obtained from the BRAD database [11] or from Phytozome [12]. Short tandem repeat sequences (STR) were identified using Tandem Repeats Finder [13]. Primers for amplification of STR were designed using Primer-BLAST [14] and tested for polymorphism between DWRCBr76 and DWRCBr57. Genetic markers usable for mapping are given in Table 1.

**Table 1:** Markers used for genetic mapping.

Marker	A09 Position^1^	Forward Primer	Reverse Primer
*D9BrapaS1*^2^	7,222,274	CCAGCCAAATCGTCACTCATGCGA	TGCATGCCTAAGAGTTTGGAGTAACAC
*D9BrapaS8*	10,306,916	AACTGAGCTTCCCATGTCCG	ACTTCTTATCTGCGTGGCAA
*D9BrapaS7*	10,989,431	CCTCTCTGGCTGATTTTGGTG	AATCCGCCGCTATGAGGAAC
*D9BrapaS9*	11,459,243	ACAGGGAGGAGGAGCAATCTT	GAGATGACTCACCCGGAATCG
*D9BrapaS10*	11,617,011	GATCGGGCATAGCCACCAAA	CACTTATAGGTGGAATGGTCACA
*D9BrapaS11*	11,877,226	AATCGACGTAGGTCTGCTGT	AAGGAAAGCTTCCCGGCAATA
*D9BrapaS15*	11,890,565	AGAGAATGAGAGGCATGGTGAA	CCTCAACCTCTAGCTTGCACT
*D9BrapaS14*	12,102,596	GAAGAAGCAGGCGATCACG	AGAGGAAAGCATGAGAGAGAGG
*D9BrapaS13*	14,111,236	CCATGTCCAGGAACAGGTTAAAA	TTACAGCAACCAACCAACAGATA
*D9BrapaS12*	19,689,771	AAGCCTAGACTTAGCAGTAAGAACA	AAGCGTCAATCGACTTCTTGC
*D9BrapaS6*	19,474,795	ATGTCCCGTTTTCGTATCCA	CTCGGAGAGCTACTGTCTGC
*D9BrapaS5*	29,261,082	AGCTGAAGCCAATCAAACTGAA	TGGAGATTAGTGTGCCCTGA
*Bn9A*^3^	30,357,828	GAGCCATCCCTAGCAAACAAG	CGTGGAAGCAAGTGAGATGAT
*D9BrapaS4*^2^	35,598,603	TCGAGCTGAGAGGGAAGCTGTGA	AGCGATGTAGCACCCGAGTCCA
*Park9HaeIII*^2^	36,389,743	TTGCGACAAAGAAACACAGC	TCCTCAGCTGCTTTAGCCTC

^1^Position of 5’ end of forward primer in *Brassica rapa* FPsc v1.3, DOE-JGI, http://phytozome.jgi.doe.gov/ [12].

^2^Source Slankster *et al* [10]

^3^Developed by Kresovich and applied to *RCBr* as reported by Slankster *et al* [10]

*D9BrapaS5* and *D9BrapaS12* are dominant markers which give a PCR product in DWRCBr76 but not in DWRCBr57.

### DNA Purification

For DNA purification, plant tissue was disrupted in a ground glass homogenizer in 500 μl of 50 mM tris base, 240 mM NaCl, 25 mM EDTA, 0.5% SDS with RNAse H. The homogenate was extracted with chloroform and isoamyl alcohol (24:1). DNA was precipitated with an equal volume of isopropyl alcohol. The pellet was washed with 70% ethanol, air dried, and dissolved in TE buffer, pH 7.5.

### Polymerase Chain Reaction (PCR)

Genomic DNA was amplified by PCR with the following protocol: 94° for 2 min., 30 cycles of 94° for 30 sec., 61° for 1 min., 72° for 1 min., and finally 72° for 4 min.

### Genotyping

For most genetic markers, alleles were resolved on 1.2% agarose gels. Markers that could not be resolved on agarose gels were resolved in nondenaturing polyacrylamide gels consisting of 8% total acrylamide (19:1 acrylamide:bis) in Tris-Borate EDTA buffer.

### Mapping by Backcross

Strains DWRCBr76 and DWRCBr57 were crossed and the F1 generation was backcrossed to DWRCBr57. BC1 seeds were sprouted on damp blotter paper under a bank of six 40W fluorescent tubes. After three to five days of constant light exposure, a total of 126 seedlings were scored for phenotype (purple or non-purple) relative to seedlings of the parental strains. Tissue was collected for DNA purification. Genetic map distances in Kosambi centimorgans were determined using MapDisto [15]. Marker order was set by their position in the *Brassica rapa* genome FPsc ver. 1.3 in Phytozome [12].

### Homozygosity Mapping

Strains DWRCBr57 and DWRCBr76 were crossed and pairs of F1 generation plants were intercrossed to produce an F2 generation. F2 seeds were sprouted as described above and only non-purple seedlings were collected for mapping. A total of 1199 F2 seedlings were produced. Of these, 402 were non-purple and used for homozygosity mapping. All 402 non-purple F_2_ seedlings (sprouted as described above) were genotyped for markers *D9BrapaS7* and *D9BrapaS6*. Individuals that were recombinant between these two markers were genotyped for additional markers within the interval.

### Molecular Cloning

PCR amplicons were purified for DNA sequencing using a MinElute PCR Cleanup Kit (Qiagen Inc., Valencia, CA). Fragments were cloned in to pGEM-T vector (Promega, Madison, WI, USA) and transformed into competent 10-beta *E*. *coli* cells (New England Biolabs, Ipswich, MA, USA).

### DNA sequencing

Sequencing reactions were performed using ABI BigDye Terminator v3.1 Cycle Sequencing Kit and analyzed using the Applied Biosystems ABI Prism 3730 DNA Analyzer at the Wayne State University Applied Genomics Technology Center (agtc.wayne.edu). Nucleotide sequences were submitted to Genbank under accessions numbers KX185527, KX347549, and KX379243.

## Results

### Fine mapping of *anthocyaninless* to proximal chromosome A09

**Fig 1:**
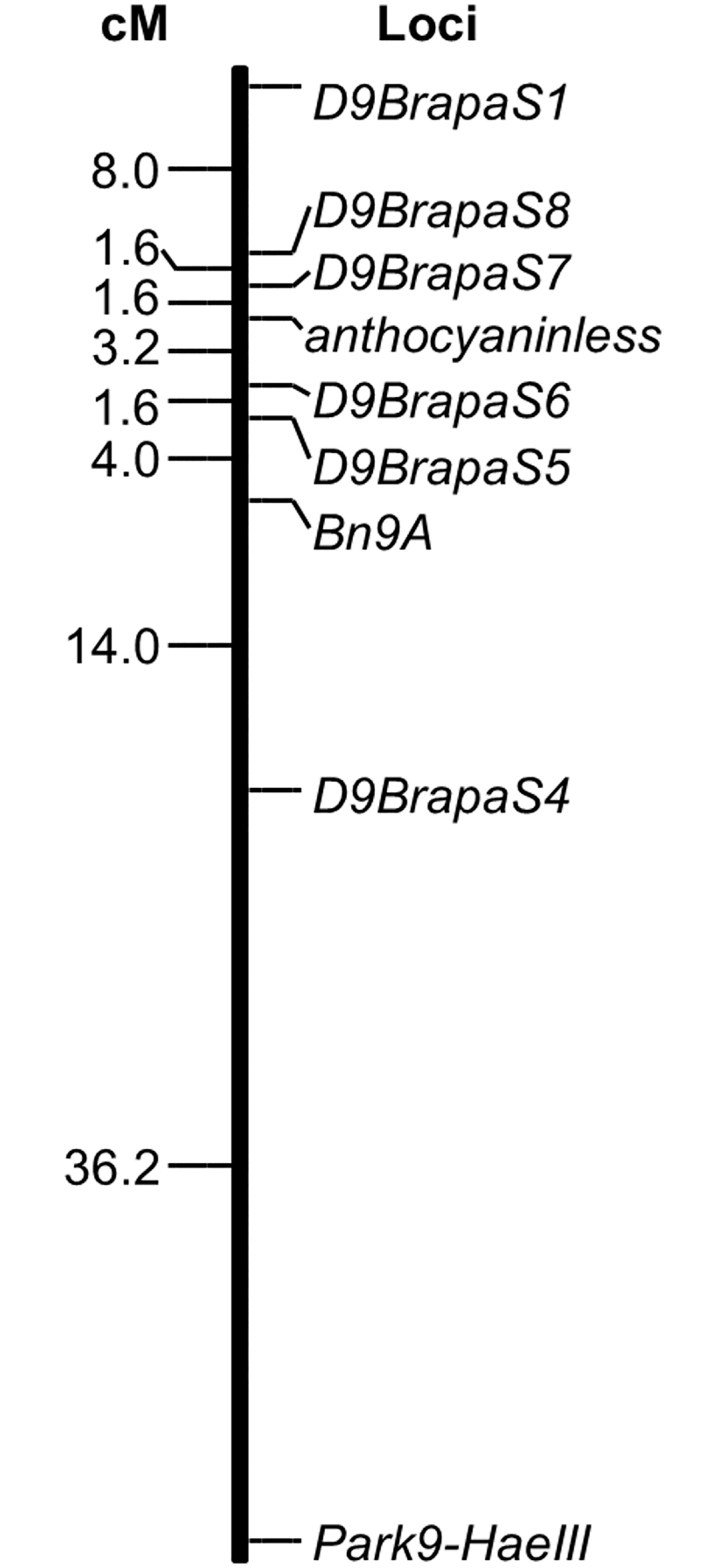
Genetic linkage map of chromosome A09 including DNA markers tightly linked to the *anthocyaninless* (*ANL*) locus. Genotype data are from 126 BC1 progeny. Distances are in Kosambi centimorgans and all linkages have a LOD > 3.0.

The *anl* locus was previously mapped to *Brassica rapa* chromosome A09 [16], but with low resolution. To more precisely map the position of *anl* we developed additional markers (Table 1) and generated larger sample sizes. In a backcross with 126 progeny, we mapped *anl* to within the interval between markers *D9Brapa6* and *D9Brapa7* (Fig 1). This interval is 3.8 centimorgans on our genetic map and 8.4 Mb in the *Brassica rapa* genome assembly *FPsc v1*.*3*.

**Fig 2:**
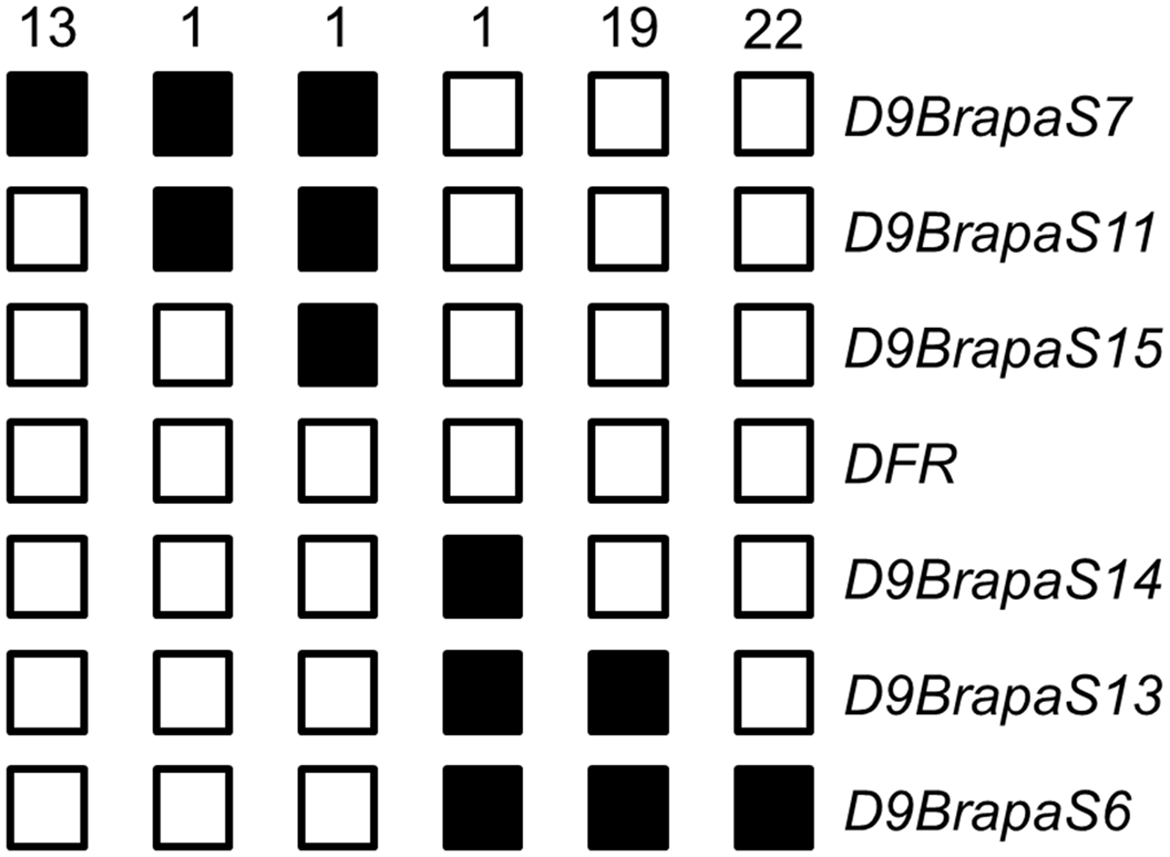
Fine mapping places the *anthocyaninless* gene to a 212 kb interval between *D9BrapaS15* and *D9BrapaS14*. 57 nonpurple F_2_ progeny (out of a total of 402 non-purple F_2_ progeny) were recombinant between *D9BrapaS7* and *D9BrapaS6*. These were then genotyped for markers within the interval. A shaded box indicates the presence of the marker allele from the purple strain DWRCBr76.

We next used homozygosity mapping to further refine the position of the *anl* gene. We genotyped 402 non-purple (*anl/anl* genotype) F_2_ generation seedlings (a total of 804 informative chromosomes) for markers *D9BrapaS6* and *D9BrapaS7*. Among these, 57 individuals were recombinant between one of the markers and the *anl* locus. Genotyping of these 57 recombinants for additional markers between *D9BrapaS6* and *D9BrapaS7* (Fig 2) allowed us to narrow the position of *anl* down to a 212 kilobase interval which contained 28 predicted genes (Table 2). Of the annotated genes in the interval, Phytozome gene *Brara*.*I01754*, which encodes dihydroflavonol 4-reductase (*DFR*) stood out as the most likely candidate because mutations of this gene have been shown to result in an anthocyaninless phenotype in at least ten other plant species across the plant kingdom [9,17–25].

**Table 2:** Genes in the interval containing *anthocyaninless*.

Gene	Annotation
Brara.I01732	auxin-inducible
Brara.I01733	none
Brara.I01734	solute carrier family 35
Brara.I01735	60S ribosomal protein L3-related
Brara.I01736	zinc finger FYVE domain containing protein
Brara.I01737	clathrin coat assembly protein
Brara.I01738	uncharacterized protein family
Brara.I01739	Agenet
Brara.I01740	Protein of unknown function, DUF617
Brara.I01741	Protein of unknown function, DUF547
Brara.I01742	Apoptosis-promoting RNA-binding protein
Brara.I01743	none
Brara.I01744	none
Brara.I01745	histone H3
Brara.I01746	uncharacterized protein ath:AT5G42710
Brara.I01747	F-box/leucine rich repeat protein
Brara.I01748	gluose-6-phosphate isomerase
Brara.I01749	zinc finger FYVE domain containing protein
Brara.I01750	uncharacterized protein ath:AT5G42765
Brara.I01751	ZF-HD protein dimerization region
Brara.I01752	uncharacterized protein ath:AT5G42785
Brara.I01753	proteasome subunit alpha/beta
Brara.I01754	dihydroflavonol 4-reductase
Brara.I01755	inositol polyphosphate kinase 1
Brara.I01756	U2 SNRNR auxiliary factor, small subunit
Brara.I01757	uncharacterized protein ath:AT5G42860
Brara.I01758	lipin 3-related
Brara.I01759	sterol carrier protein 2
Brara.I01760	RNase_H

### Mutation in the *DFR* gene

**Fig 3:**
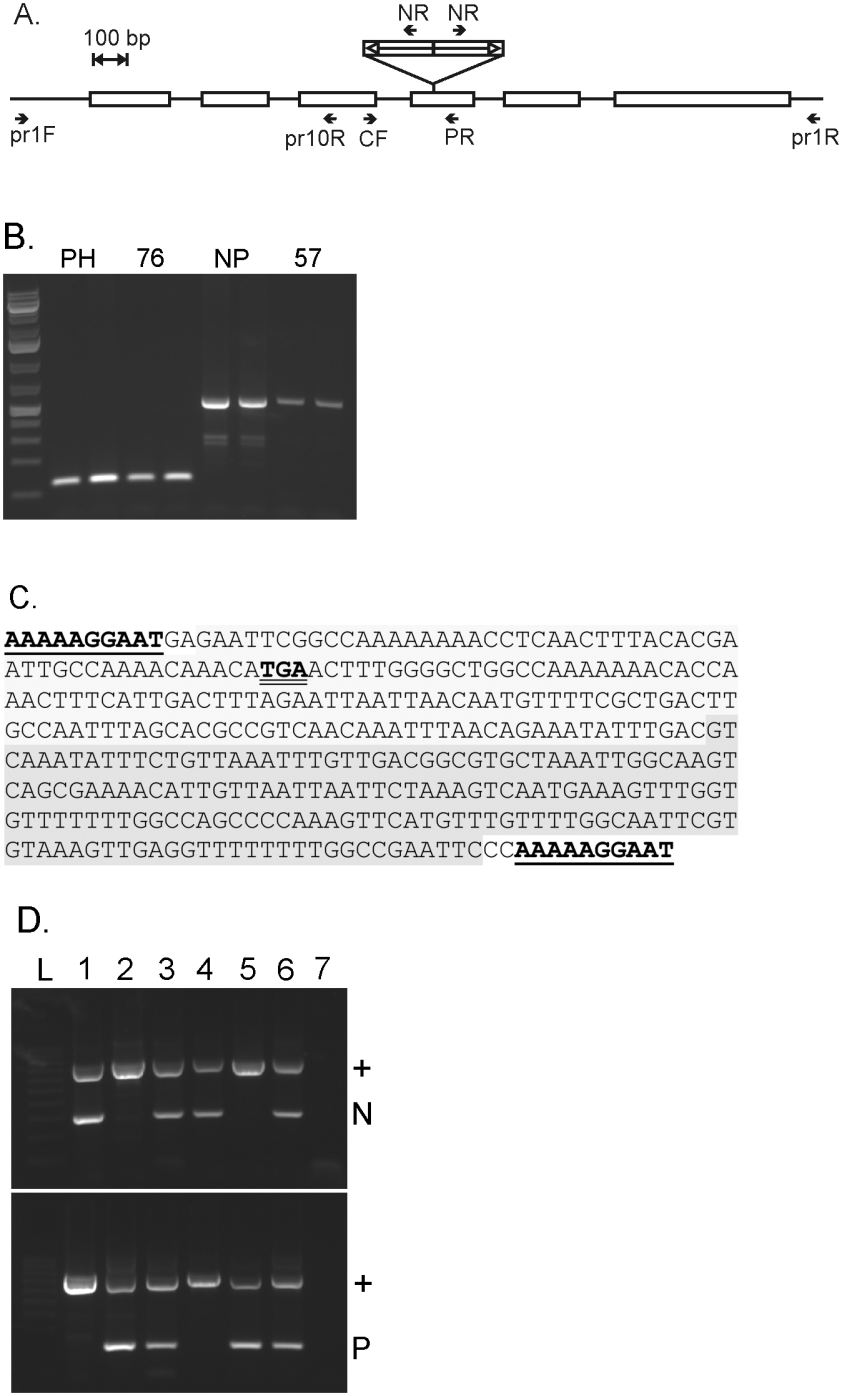
Non-purple strains bear an insertion mutation in exon 4 of the *DFR* gene. (A) Diagram of predicted intron/exon structure of *DFR* with the insertion shown. Positions of primers used in this study are also shown. (B) PCR with primers pr7F and PR which are anchored in the 5^th^ exon of the gene identifies an insertion mutation. (C) Nucleotide sequence of the insertion (GenBank accession KX379243). Flanking direct repeats are underlined. The two halves of the perfect palindrome are shaded. An in-frame stop codon is indicated with double underline. (D) PCR assay for wild type and mutant alleles of the *DFR* gene. In the top panel, PCR primers were prCF and prNR which amplify a product (N) in alleles with the insertion. In the bottom panel, PCR primers were prCF and prPR which amplify a product (P) from the wild type allele. In all reactions, primers pr1F and pr10R, which amplify a segment from a different part of the *DFR* gene (+), were included to serve as a positive control. The DNA samples used were (1) Non-purple Stem Yellow Green Leaf, (2) Purple Hairy, (3) hybrid of 1 and 2, (4) DWRCBr57, (5) DWRCBr76, (6) hybrid of 4 and 5, (7) no template control.

Analysis of the *DFR* gene in purple and nonpurple strains revealed that the nonpurple strains have an insertion mutation in the fourth exon of the *DFR* gene (Fig 3). PCR reactions with primers anchored in exon 4 (Table 3) of the gene indicated an insert of 300–400 bp (Fig 3-B).

**Table 3:** Primers used to characterize the *DFR* gene.

Primer	Sequence
pr1F	CGAGCCAACGCACATTTCAT
pr1R	TCCACGTGACCGTAAATCCT
pr7F	TAGCCGAGAAAGCAGCTTGG
pr10R	TCTTCGTACGGTCTTTGCCT
prCF	CCAAGAAGATGACAGGATGGGT
prNR	TTGACGGCGTGCTAAATTGG
prPR	TGGACCGATCACCAATGTCG

We cloned genomic DNA of the *DFR* gene from wild type strains and mutant strains for nucleotide sequencing. Alignment of the nucleotide sequences of the *DFR* gene from wild type strains with the predicted mRNA of Phytozome gene *Brara*.*I01754* indicated that the purple strains have a functional *DFR* gene sequence. Initial attempts to sequence the mutant allele of the gene failed at a point roughly in the middle of predicted exon 4. This suggested a sequence that was forming a secondary structure such as a hairpin that is refractory to the sequencing reaction. Therefore we subcloned separate sections of the insert for sequencing. We screened a collection of restriction enzymes and found that one, *HpyCH4IV*, divided the segment approximately in half. We subcloned each half and were successful at sequencing each half separately. Assembly of these sequences revealed that the insertion sequence has a 340 bp sequence consisting of an inverted repeat with two 170 bp halves that form a perfect palindrome. The inverted repeat was flanked by direct terminal repeats (Fig 3-C). To verify that this sequence was not a cloning artifact, we designed a PCR primer that was anchored in the insert and designed to prime away from the center of the palindrome and used this to test for the existence of the palindrome in genomic DNA samples from the nonpurple strains. When the insert-specific primer was paired with a primer from the gene sequence upstream of the insert, it produced a PCR product. The insert-specific primer also produced a PCR product when paired with a primer from the gene sequence downstream of the insert (Fig 3-D). This confirmed the structure indicated by the DNA sequencing data.

The insertion introduces a premature stop codon into the gene. There is TGA beginning at the 53^rd^ base of the palindrome which is in frame when aligned with the predicted mRNA of *Brara*.*I01754* (Fig 3-C).

The presence of flanking direct repeats suggested a transposition event. A BLAST search of the *Brassica rapa* genome using the inserted sequence did not identify any other palindromic sequences, but it did identify over 100 sequences with greater than 90% identity and 90% length equivalent to one “half palindrome”. Of all of the BLAST hits in *Brassica* the longest were 170 base pairs. We did not find any other examples of the inverted repeat arrangement in either the *FPsc v1*.*3* genome in Phytozome [12] or Chiifu-401 genome in BRAD [11]. A similar result was found in *Brassica olearacea*, but no such sequence was found in other species, including *Arabidopsis thaliana*. Thus the insertion in *DFR* found in non-purple rapid cycling *Brassica rapa* appears to be specific to its genus.

### Complete linkage between *anl* and *DFR*

To confirm the identity of the *DFR* gene as the *anthocyaninless* locus, we genotyped the BC_1_ and F_2_ groups described above for the mutant and wild type alleles of *DFR*. Together these groups provide 930 informative chromosomes but there were no recombinants between *DFR* and *anl*. We also genotyped 18 non-purple F_2_ progeny from a cross of Purple Hairy and Non-purple Stem, Yellow Green Leaf. These were also homozygous for the mutant allele of *DFR*.

### Some purple plants have an insertion mutation in the last intron

**Fig 4:**
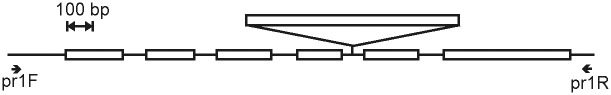
Purple strain DWRCBr76 bears an insertion within the intron between exons 5 and 6 of the *DFR* gene (GenBank accession KX347549).

Alignment of genomic DNA sequences of the DFR gene from the two purple strains Purple Stem, Hairy and DWRCBr76 revealed that the DWRCBr76 strain carries a 754 bp insertion in the intron between exons 4 and 5 of the *DFR* gene (Fig 4).

### A simple test for the *anl* mutation suitable for instructional labs

**Fig 5:**
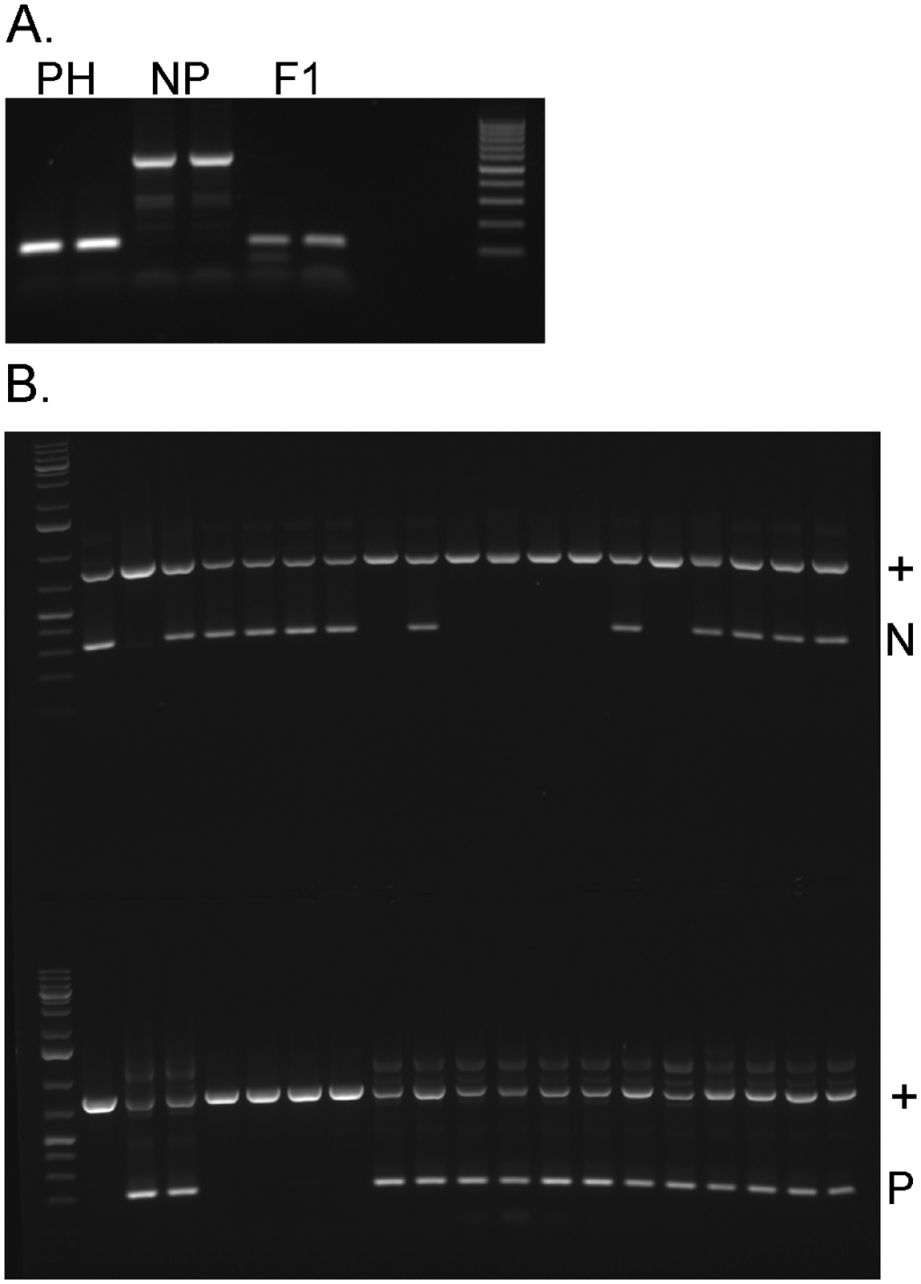
Development of a PCR test of the *anl* mutation for the teaching laboratory. (A0 When DNA from a heterozygote is used as PCR template, wild type and mutant alleles cannot be simultaneously amplified. Gel contains products of duplicate reactions of Purple Stem, Hairy (PH), Nonpurple Stem, Yellow Green Leaf (NP), and a hybrid of the two (F1). (B) A PCR test that detects the mutant (N) and wild type (P) alleles in separate reactions in plants grown from Carolina Biological Supply seeds. PCR is as described in Fig 4.D. Lanes: (1) DNA ladder, (2) Nonpurple Stem, Yellow Green Leaf, (3) Purple Hairy, (4) F_1_ generation, (5–8) F_2_ seedlings with nonpurple phenotype, (9–20) F_2_ seedlings with purple phenotype.

With the PCR described for Fig 4-C one combination of primers specifically detects the mutant allele and another detects the wild type allele. Normally, to detect an insertion or deletion by PCR one simply uses a single pair of primers and observes a change in the size of the PCR product, and a heterozygote is detected as a double band. However, when we perform such a PCR with an *ANL/anl* heterozygote the wild type allele amplifies preferentially (Fig 5-A), presumably due to the palindromic insert reducing PCR efficiency. Therefore, each allele must be detected in separate PCR reactions. This strategy makes a very robust and clear PCR assay for the mutant allele of the gene that can be used in an undergraduate or advanced high school teaching lab. Fig 5 shows the results of genotyping seedlings from stocks obtained from Carolina Biological Supply. The mutant and wild type alleles are readily detected in parental strains. In a sample of F_2_ generation seedling the PCR test shows that all nonpurple seedlings have the mutant allele only, and among the purple seedlings, some are have only the wild type allele and some carry the mutant allele in addition to wild type.

## Discussion

Our data indicate that the *DFR* gene which encodes dihydroflavonol 4-reductase is the *anthocyaninless* gene of Rapid Cycling *Brassica rapa* (Fast Plants type). The non-purple trait maps to this position and the *DFR* gene on non-purple strains bears an insertion mutation that would doubtlessly inactivate the gene. If the palindromic sequence does not impede transcription and translation, it introduces a premature stop codon in the fourth exon of the gene. The linkage of loss of function mutation in *DFR* to the *anthocyaninless* locus is consistent with what is seen in other plant species. *DFR* encodes an enzyme that is essential for flavonol biosynthesis, and loss of function mutations have been found to result in a complete lack of anthocyanin pigments in maize [17], snapdragon [18], mouse ear cress [19], tomato [20], soybean [9], tobacco [21], Lewis’ monkeyflower [22], onion [23], Solonaceae [24], and Oriental lily [25].

Both nonpurple strains analyzed in this study have the insertion mutation. That is to be expected because although there are multiple nonpurple seed stocks of RCBr, they originate from the same original isolate (P. Williams, personal communication).

Educators at the advanced high school and introductory college level can employ a simple PCR assay to determine a plant’s genotype for *DFR* and relate this to a plant’s phenotype.

Our work identifying the mutation responsible for the non-purple trait will be of great value for educators using Fast Plants for genetics instruction. Our findings are the first case of a trait in Fast Plants being characterized at the molecular level, and the recessive non-purple trait (genotype *anl*/*anl*) is the principle trait used in Mendelian genetics exercises developed by the Wisconsin Fast Plants Program [3]. Now the nonpurple trait is an observable phenotypic variant can be used as a gateway to genomics. The genome of *Brassica rapa* has been sequenced in both Chinese cabbage [26] and in the FPSc strain of self-compatible Fast Plants [27]. Both can be searched and browsed in public databases. Students who study classical genetics with crosses of purple and nonpurple will now be able to relate this observable phenotypic variation to variation in a specific sequence and explore the genomic sequence data and browse the chromosome around the *DFR* gene. It is also a gateway to connecting an easily observable trait, purple stem color, with a major biochemical pathway, flavonoid biosynthesis.

## References

[1] Tomkins S, Williams P. Fast Plants for Finer Science—an Introduction to the Biology of Rapid-Cycling Brassica-Campestris (Rapa) L. J Biol Educ. 1990;24: 239–250.

[2] Lauffer HB, Lauffer D, Williams P. Wisconsin Fast Plants Program.2012.

[3] WISCONSIN FAST PLANTS PROGRAM. Wisconsin Fast Plants Manual. Burlington, NC, USA: Carolina Biological Supply; 2016.

[4] Goldman IL. Teaching Recurrent Selection in the Classroom with Wisconsin Fast Plants.1999;9: 579–584.

[5] Batzli JM, Smith AR, Williams PH, Mcgee SA, Dosa K, Pfammatter J. Beyond Punnett Squares: Student Word Association and Explanations of Phenotypic Variation through an Integrative Quantitative Genetics Unit Investigating Anthocyanin Inheritance and Expression in Brassica rapa Fast Plants. CBE-Life Sci Educ. 2014;13: 410–424. 10.1187/cbe.13-12-0232 25185225 PMC4152203

[6] Grotewold E. The genetics and biochemistry of floral pigments.2006;57: 761–780. 10.1146/annurev.arplant.57.032905.105248 16669781

[7] Gould K, Davies KM, Winefield C, editors. Anthocyanins: Biosynthesis, Functions, and Applications. New York: Springer; 2014.

[8] Holton T, Cornish E. Genetics and Biochemistry of Anthocyanin Biosynthesis. Plant Cell. 1995;7: 1071–1083. 12242398 10.1105/tpc.7.7.1071 PMC160913

[9] Xu M, Brar HK, Grosic S, Palmer RG, Bhattacharyya MK. Excision of an Active CACTA-Like Transposable Element From DFR2 Causes Variegated Flowers in Soybean [Glycine max (L.) Merr.]. Genetics. 2010;184: 53–U110. 10.1534/genetics.109.107904 19897750 PMC2815930

[10] Slankster EE, Chase JM, Jones LA, Wendell DL. DNA-Based Genetic Markers for Rapid Cycling Brassica Rapa (Fast Plants Type) Designed for the Teaching Laboratory.2012;3: 118. 10.3389/fpls.2012.00118 PMC3365483 22675329

[11] Cheng F, Liu S, Wu J, Fang L, Sun S, Liu B, et al BRAD, the genetics and genomics database for Brassica plants.2011;11: 136. 10.1186/1471-2229-11-136 PMC3213011 21995777

[12] Goodstein DM, Shu S, Howson R, Neupane R, Hayes RD, Fazo J, et al Phytozome: a comparative platform for green plant genomics. Nucleic Acids Res. 2012;40: D1178–86. 10.1093/nar/gkr944 22110026 PMC3245001

[13] Benson G. Tandem repeats finder: a program to analyze DNA sequences. Nucleic Acids Res. 1999;27: 573–580. 9862982 10.1093/nar/27.2.573 PMC148217

[14] Ye J, Coulouris G, Zaretskaya I, Cutcutache I, Rozen S, Madden TL. Primer-BLAST: a tool to design target-specific primers for polymerase chain reaction. BMC Bioinformatics. 2012;13: 134 10.1186/1471-2105-13-134 22708584 PMC3412702

[15] Loreiux M. MapDisto: fast and efficient computation of genetic linkage maps.2012;30: 1231–1235.

[16] Burdzinski C, Wendell DL. Mapping the Anthocyaninless (anl) locus in rapid-cycling Brassica rapa (RBr) to linkage group R9. BMC Genet. 2007;8: 64 17894874 10.1186/1471-2156-8-64 PMC2048511

[17] Reddy AR, Britsch L, Salamini F, Saedler H, Rohde W. The A1 (anthocyanin-1) locus in Zea mays encodes dihydroquercetin reductase.1987;52: 7–13.

[18] Beld M, Martin C, Huits H, Stuitje AR, Gerats AGM. Flavonoid Synthesis in Petunia-Hybrida—Partial Characterization of Dihydroflavonol-4-Reductase Genes. Plant Mol Biol. 1989;13: 491–502. 2491667 10.1007/BF00027309

[19] Shirley BW, Hanley S, Goodman HM. Effects of Ionizing-Radiation on a Plant Genome—Analysis of 2 Arabidopsis Transparent-Testa Mutations. Plant Cell. 1992;4: 333–347. 1354004 10.1105/tpc.4.3.333 PMC160133

[20] Goldsbrough A, Belzile F, Yoder JI. Complementation of the Tomato Anthocyanin without (Aw) Mutant using the Dihydroflavonol 4-Reductase Gene. Plant Physiol. 1994;105: 491–496. 12232217 10.1104/pp.105.2.491 PMC159386

[21] Kazama Y, Fujiwara MT, Takehisa H, Ohbu S, Saito H, Ichida H, et al Characterization of a heavy-ion induced white flower mutant of allotetraploid Nicotiana tabacum. Plant Cell Rep. 2013;32: 11–19. 10.1007/s00299-012-1336-7 22930364

[22] Wu CA, Streisfeld MA, Nutter LI, Cross KA. The Genetic Basis of a Rare Flower Color Polymorphism in Mimulus lewisii Provides Insight into the Repeatability of Evolution.2013;8: e81173. 10.1371/journal.pone.0081173 PMC3849174 24312531

[23] Song S, Kim C, Moon JS, Kim S. At least nine independent natural mutations of the DFR-A gene are responsible for appearance of yellow onions (Allium cepa L.) from red progenitors. Mol Breed. 2014;33: 173–186.

[24] Coburn RA, Griffin RH, Smith SD. Genetic Basis for a Rare Floral Mutant in an Andean Species of Solanaceae. Am J Bot. 2015;102: 264–272. 10.3732/ajb.1400395 25667079

[25] Suzuki K, Tasaki K, Yamagishi M. Two distinct spontaneous mutations involved in white flower development in Lilium speciosum. Mol Breed. 2015;35: 193.

[26] Wang X, Wang H, Wang J, Sun R, Wu J, Liu S, et al The genome of the mesopolyploid crop species Brassica rapa. Nat Genet. 2011;43: 1035–1039. 10.1038/ng.919 21873998

[27] Woody S. FPsc: A New, Plant-Based Model System for Integrated Education in Genetic and Genomic Sciences. 2016: W800.

